# Brain-Computer Interface to Deliver Individualized Multisensory Intervention for Neuropathic Pain

**DOI:** 10.1007/s13311-023-01396-y

**Published:** 2023-07-05

**Authors:** Giuseppe Valerio Aurucci, Greta Preatoni, Arianna Damiani, Stanisa Raspopovic

**Affiliations:** Laboratory for Neuroengineering, Department of Health Science and Technology, Institute for Robotics and Intelligent Systems, ETH Zürich, 8092 Zurich, Switzerland

**Keywords:** Neuropathic pain, Transcutaneous electrical nerve stimulation, Virtual reality, EEG, Skin conductance, BCI

## Abstract

To unravel the complexity of the neuropathic pain experience, researchers have tried to identify reliable pain signatures (biomarkers) using electroencephalography (EEG) and skin conductance (SC). Nevertheless, their use as a clinical aid to design personalized therapies remains scarce and patients are prescribed with common and inefficient painkillers. To address this need, novel non-pharmacological interventions, such as transcutaneous electrical nerve stimulation (TENS) to activate peripheral pain relief via neuromodulation and virtual reality (VR) to modulate patients’ attention, have emerged. However, all present treatments suffer from the inherent bias of the patient’s self-reported pain intensity, depending on their predisposition and tolerance, together with unspecific, pre-defined scheduling of sessions which does not consider the timing of pain episodes onset. Here, we show a Brain-Computer Interface (BCI) detecting in real-time neurophysiological signatures of neuropathic pain from EEG combined with SC and accordingly triggering a multisensory intervention combining TENS and VR. After validating that the multisensory intervention effectively decreased experimentally induced pain, the BCI was tested with thirteen healthy subjects by electrically inducing pain and showed 82% recall in decoding pain in real time. Such constructed BCI was then validated with eight neuropathic patients reaching 75% online pain precision, and consequently releasing the intervention inducing a significant decrease (50% NPSI score) in neuropathic patients’ pain perception. Our results demonstrate the feasibility of real-time pain detection from objective neurophysiological signals, and the effectiveness of a triggered combination of VR and TENS to decrease neuropathic pain. This paves the way towards personalized, data-driven pain therapies using fully portable technologies.

**Supplementary Information:**

The online version contains supplementary material available at 10.1007/s13311-023-01396-y.

## Introduction

Chronic neuropathic pain is a distressing health problem affecting up to 6.9% of the general population [1] which is caused by a lesion or dysfunction of the nervous system [2]. Patients typically report burning, shooting, or electric-like sensations [3–5], which can be episodic or constant. Moreover, this phenomenon is strongly influenced by emotional and cognitive aspects [6–9]. The overlap and interconnection between these components lead to a vicious cycle: being in a chronic pain state negatively impacts the patients’ emotional sphere and may lead to depression and anxiety [10], which in turn makes patients feel helpless and catastrophize their condition [11]. As a result, pain perception becomes both the cause and the effect of the worsening of this condition. Given its complexity and subjectivity, performing a thorough and reliable pain assessment remains challenging. The gold standard for pain measurement is self-reported pain scales, such as the Numerical Pain Rating Scale (NPRS) or the Visual Analogue Scale (VAS). However, these tools fail to capture the multidimensionality of pain and are highly influenced by the patient’s suggestibility. This is driving the scientific community to search for and identify reliable neurophysiological biomarkers to objectively measure pain. Researchers are employing measures of autonomic nervous system activation, such as skin conductance (SC), to objectively quantify the arousal resulting from the pain experience [12]. SC has shown promising results for decoding noxious stimulations [13–16], as well as evaluating prolonged pain conditions (e.g., post-operative [17] pain and chronic pain [18–20]). However, SC could fail in distinguishing arousal caused by nociception or by other salient stimuli (i.e., threat), and it is therefore crucial to combine it with another neurophysiological signal to improve the detection performances. Diverse approaches for brain investigation, such as electroencephalography (EEG), functional magnetic resonance (fMRI), PET, and NIRS, are being employed to unravel unequivocal indicators of pain perception and processing [21]. Among them, EEG has the advantage of having extremely good temporal resolution and being portable, which makes it particularly suitable for real-time applications. The majority of studies conducted on healthy subjects agreed on a significant reduction of alpha power (8–13 Hz) [22–26] and increased gamma activity (> 30 Hz) [25, 27, 28] as possible biomarkers of induced pain. However, the search for pain biomarkers in the chronic population remains challenging [29, 30]. Recently, Mussigmann et al. [31] performed a comprehensive review of chronic neuropathic pain, highlighting increased theta (4–7 Hz) and high beta power (20–30 Hz), but decreased high-alpha-low-beta band (10–20 Hz) as possible neuropathic pain biomarker. Nevertheless, the search for objective biomarkers is far from being completed and their practical use in clinical settings remains scarce [32]. This lack of unequivocal pain signatures prevents the possibility of designing personalized therapies. As of today, the standard of care is the pharmacological approach, thus the usage of anticonvulsants and opioids [2, 33, 34]. Nevertheless, these are often ineffective, as evidenced by the high rates (78%) of treatment dissatisfaction [35], the persistence of pain over time, and the large placebo response [33]. Moreover, opioids carry numerous side effects [36] such as constipation, nausea, somnolence, and respiratory depression which inevitably compromise the therapy’s efficacy. This prompts the emergence of novel non-pharmacological treatments for neuropathic pain. Among these, apart from invasive solutions [37, 38], transcutaneous electrical nerve stimulation (TENS) targets the sensory component of pain by selectively activating large diameters non-nociceptive fibers [39] to activate inhibitory interneurons within the spinal cord. These prevent the noxious information from being processed by the central nervous system (Gate Control Theory [40]). Recently, virtual reality (VR) emerged as an encouraging technology to directly interact with the emotional and attentional sphere of the subject, which play a key role in the processing of pain [7, 9, 10]. With this regard, the immersive and technological features of VR allow it to be an asset in modulating the perception of pain [41]. However, since every subject has their own specific pain experience, characterized by different weights given to the sensory and emotional spheres, a thorough therapy should take this into account to provide benefits on multiple levels. In this view, even though a combination of the two aforementioned non-pharmacological approaches could result in an intervention targeting the physiological component of pain (i.e., with TENS) and at the same time modulating patients’ attention and emotion (i.e., with VR), its empirical examination remains unexplored*.* Furthermore, the experimental protocols are extremely heterogeneous in terms of time and duration of treatment, frequency, and intensity. As a consequence, the effectiveness of these approaches remains an undecided issue [42, 43]. In addition to all these limitations, all current therapeutic solutions do not account for the temporally locked release of the therapy with the pain episodes, hence negatively impacting on dosages and efficacy due to over or under-treatment [32, 44]. A closed-loop system would allow to provide the therapy in real time based on objective and measurable pain biomarkers. The need for a closed-loop system is even more crucial when the verbal information is not present (disabled person or paralyzed patients), to establish a neurophysiological data-driven communication channel [45].

To address these problems, we purposely developed a Brain-Computer Interface (BCI) detecting pain in real time through neurophysiological recordings of EEG and SC to deliver a novel multisensory intervention combining VR and TENS.

## Methods

### Subjects

Eighteen healthy subjects (10 males and 8 females, mean age of 25 ± 2) were enrolled to test and validate the system feasibility, while nine patients affected by chronic neuropathic pain (7 males and 2 females, mean age of 71 ± 8) participated in the BCI proof-of-concept study. The patients were diagnosed by their neurologist with painful diabetic peripheral neuropathy (8/9) or pain caused by idiopathic neuropathy (1/9). A more detailed characterization of the clinical sample is in Table 1. All participants signed a consent form. The study was approved by the Kantonale Ethikkommission of Zurich (Nr. 2021–02258) and was conducted in accordance with the Helsinki Declaration of 1975, as revised in 2000 (World Medical Association Declaration of Helsinki 2000). The ClinicalTrials.gov Identifier is NCT05483816.

**Table 1:** Characterization of the clinical sample. *PDPN* painful diabetic peripheral neuropathy

	**Age**	**Height** **(cm)**	**Weight** **(kg)**	**BMI**	**Medical condition**	**Time since clinical diagnosis**	**Pain type**
**P1**	77	175	75	24.49	PDPN	15	Chronic
**P2**	61	180	82	25.31	PDPN	11	Chronic
**P3**	59	190	111	30.75	PDPN	13	Chronic
**P4**	82	151	59	25.88	PDPN	9	Chronic
**P5**	70	189	96	26.88	PDPN	4	Chronic
**P6**	78	180	80	24.70	PDPN	16	Chronic
**P7**	65	180	74.3	22.93	PDPN	40	Chronic
**P8**	73	172	70	23.67	Idiopathic neuropathy	-	Chronic
**P9**	77	180	90	27.78	PDPN	10	Chronic

### Intervention Outline and Validation

**Fig. 1:**
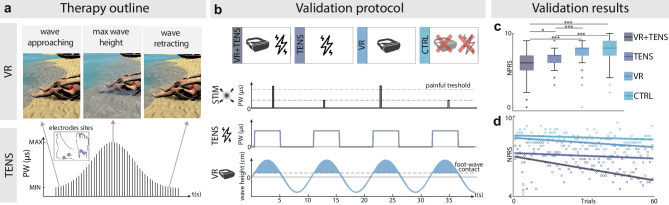
Therapy outline and validation. **a** Therapy outline. In the upper panel, a visual representation of the virtual wave moving toward the feet of the subject. In the lower panel, the TENS stimulation pattern targeting the tibial and the peroneal nerves. The stimulation pulse width follows the movement of the wave: it starts when there is contact between the wave and the feet (wave approaching), reaches its maximum (max wave height), and stops when the wave retracts (wave retracting). **b** Therapy validation protocol. Subjects receive painful and non-painful stimulations under four different experimental conditions: synchronous combination of VR and TENS (VR + TENS), TENS only (TENS), VR only (VR), and control condition (CTRL). **c** Boxplots of reported Numerical Pain Rating Scale (NPRS) following painful stimulations among subjects (*N* = 5) for each experimental condition. (* *p* < 0.05, ** *p* < 0.01, *** *p* < 0.001). **d** Changes in reported NPRS following painful stimulations over trials among subjects (*N* = 5) for each experimental condition

A RehaMove3 (Hasomed GmbH) TENS device was employed to deliver biphasic pulses at 50 Hz frequency targeting the tibial and peroneal nerves (Fig. 1a), thus inducing a spread sensation in the plantar and dorsal side of the foot respectively. The VR environment (UNITY 3D, Unity Technologies) showed a relaxing white sand beach where the subjects saw themselves from a first-person perspective as seated on a deckchair in front of the sea (Fig. 1a). The intervention was initialized by the wave movement towards the feet of the subjects; as soon as the virtual feet were in contact with the wave, the TENS was delivered [46]. The stimulation amplitude was constant, while the pulse width was modulated [46]. It varied as a Gaussian course following the height of the wave to increase the vividness of the illusion. The tactile feedback was interrupted as soon as there was no longer contact between the wave and the feet (Fig. 1a). The visuo-tactile stimulation was therefore synchronous in time and spatially congruent.

We performed an experimental validation of the intervention (Fig. 1a) on five healthy subjects (2 males, 3 females) to assess the benefits on pain perception. To this extent, TENS parameters were individually calibrated for each subject (see Supplementary Section: Intervention calibration procedure). Subjects underwent four different conditions: (1) synchronous combination of VR and TENS (intervention), (2) TENS only (Control 1), (3) VR only (Control 2), (4) control condition with nothing applied (Control 3) (Fig. 1b). During each of these conditions, subjects received 100 ms electrical pulses at the foot dorsum through an additional couple of electrodes eliciting painful (P) and non-painful (NP) sensations (see Supplementary Section: Pain calibration procedure). For each condition, subjects received 60 P and 20 NP stimuli and evaluated the perceived pain intensity on an NPRS.

### Brain-Computer Interface System

**Fig. 2:**
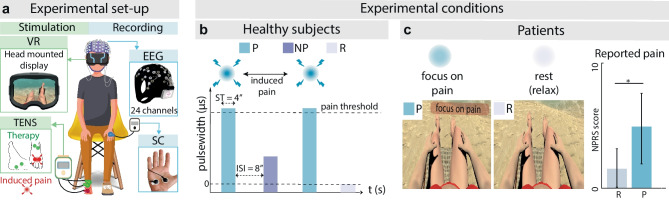
Experimental set-up and conditions. **a** Experimental set-up. Stimulation: the subject is sitting on a chair and is equipped with a head-mounted display; the electrical stimulator delivers TENS therapy targeting peroneal and tibial nerves (green electrodes); for healthy subjects, a third channel is employed for pain induction (red electrodes). Recording: electroencephalography (EEG) and skin conductance (SC) are recorded. **b** Experimental protocol for healthy subjects. Healthy subjects receive painful (blue, P), non-painful (purple, NP), and no stimulation (gray, R). **c** Experimental protocol for patients. Patients are asked to focus on their painful limb (blue, P) or to rest (gray, R). On the right, NPRS reported by patients (*N* = 8) for conditions P and R (mean ± SD). ST, stimulation time; ISI, inter-stimulus interval

Once we validated the intervention, we built and tested the Brain-Computer Interface to event-lock the release of the intervention based on EEG and SC signatures of pain. We used a 24-channels portable EEG device (SMARTING MOBI, mBrain Train) with a sampling frequency of 500 Hz, and an eSense MINDFIELD sensor for SC with a sampling frequency of 5 Hz (Fig. 2a). Online data processing was performed in Python, where EEG and SC signals were streamed through Lab Streaming Layer (LSL) and Open Sound Control (OSC) communication protocols respectively.

### Healthy Subjects’ Protocol

Firstly, the EEG cap was placed on the subject’s head. For each of the electrodes, a scrubbing procedure followed by the spreading of high chloride abrasive electrolyte gel was performed to ensure low skin–electrode impedances (< 10 KOhm). Simultaneously, the SC electrodes were placed on the subject’s left-hand palm. Afterward, given the crucial importance of an optimal calibration to elicit intuitive and electro-tactile widespread sensation [47, 48], three different calibration procedures (Fig. S1) were performed to find the individual parameters of the TENS intervention, together with the parameters for painful (P) and non-painful (NP) conditions. Subjects sat on a chair and wore the VR headset (Fig. 2a) during two offline recordings. They were asked to remain as still as possible to minimize the presence of artifacts and to keep their eyes open. For each of the recordings, subjects received in a randomized order twelve painful (P) stimulations, non-painful (NP) stimulations, and no stimulations (Rest-R). The stimulation time (ST) was 4 s, with an inter-stimulus interval (ISI) of 8 s (Fig. 2b). Between the two recordings, another pain calibration procedure was performed to avoid adaptation to the electrical stimulation. For each subject, the collected EEG and SC data were processed to train two ML models decoding pain, for EEG and SC respectively. The BCI detection framework performances were then tested online while subjects received randomized P, NP, and R stimuli for an overall time of 10 min.

### Healthy Subjects’ Offline Neurophysiological Signal Processing

EEG data collected during offline recordings was band-pass (3–40 Hz) filtered through an FIR windowed (Hamming) sinc filter of order 2000. Six electrodes in the centro-parietal area were then selected (Cz, C3, C4, CPz, CP1, CP2) to focus the analysis on the central area of the somatosensory cortex, known as one of the crucial areas involved in pain processing. Within each of the 4-s stimuli duration, Running Observation Windows (ROW) of 500 ms with 80% overlap were extracted and labeled according to the epochs from which they were taken (P, NP, R). The choice of 500 ms ROW ensured a reasonable compromise between adequate frequency resolution and loss of signal stationarity [49]. Windows with peak-to-peak amplitude over 150 μV were rejected as artifacts. From each of the windows, frequency and entropy features were extracted (Tables 2 and S1). For frequency features, the power spectral density of each 500 ms window (250 samples) was estimated with fast Fourier transform allowing zero padding to increase the number of samples up to 1024. Features were *z*-scored and then fed into a support vector machine (SVM) Classifier with Radial Kernel (*C* = 1 and *γ* = 0.01), which was employed due to its suitability for BCI applications [50]. Offline performances were evaluated with a four fold cross-validation both for 3-classes (P, NP, R) and 2-classes (P, R). Due to the high inter-subject variability of pain perception and response [51], we trained the models to decode pain specifically for each subject. The cross-validation outline guaranteed that observation within the same P, NP, or R stimuli was in different folds to avoid possible overfitting caused by overlapping. It is of utmost importance for our system to detect pain with a limited delay to deliver the intervention in real time. Therefore, even though SC spectral content is at lower frequencies than EEG (from 0 to 2 Hz), a short ROW (2 s with 80% overlap) was taken for features extraction. The signal was then band-passed between 0.05 and 2 Hz (Chebyshev type I, order 3) to highlight the stimulus-induced phasic response. Windows with peak-to-peak amplitude > 10µS were discarded since such variation within a 2-s window may indicate the dropping of one of the electrodes. Features were extracted (Tables 2 and S1), standardized, and fed into a radial kernel SVM (*C* = 1 and *γ* = 0.01). Offline performances were again evaluated with a four fold cross-validation both for 3-classes and 2-classes outlines, following the same method described for EEG.

**Table 2:** EEG (left column) and SC (right column) features

***EEG Features***	***SC Features***
Theta Power	Mean
Alpha Power	Max
Beta Power	Median
Gamma Power	SD
0-40 Hz Power	VAR
Peak frequency	IQR
Sample entropy	RMS
Spectral entropy	Range
Higuchi Fractal Dimension	Slope
RMS	MAD
	AUC

### Healthy Subjects’ BCI Online Implementation

For the online phase, the EEG and SC 2-classes trained models were loaded. Since EEG and SC work on two different classifiers (due to different communication protocols), we implemented a pipeline to merge information from both signals. Here, 500 ms EEG and 2 s SC chunks are processed to produce a classification every 500 ms each. The final classification is made every second and relies on the last 4 classifications according to a probabilistic approach (see Supplementary Section: Probabilistic approach). To evaluate the online performances of the BCI detection framework we considered a painful stimulation (P) as correctly classified if the BCI released the intervention within 4 s from the end of the stimulation; non-painful stimulation (NP) and no stimulation (R) as correctly classified if the BCI did not release the intervention within 4 s from the end of the stimulation.

### Patients’ Protocol

We tested the Brain-Computer Interface also on eight neuropathic patients (due to time constraints, one patient completed the offline phase only) by slightly changing some of its characteristics due to the intrinsically different nature and manifestation of their pain. Patients were asked to fill out the Neuropathic Pain Symptom Inventory (NPSI) [52] the morning of the experiment (before the session) (Table S5) and the day after. The characteristics of pain in terms of intensity, quality, and time were assessed with the NPSI. As reported in Q4 and Q7 of the NPSI, all patients reported permanent pain and at least between 1 and 5 pain attacks in the last 24 h (Table 3). Given the unfeasibility of waiting for the patient to experience a pain attack while wearing EEG and SC to train the BCI, we slightly adapted the patients’ experimental protocol. To ensure a more consistent and uniform protocol among patients and to favor a scenario where the patients perceived a higher level of pain, we trained the classification model depending on whether the patients were focusing or not focusing on pain. Indeed, attention plays a crucial role in the modulation of pain [7, 10, 53] and it has been shown that focusing on pain positively correlates with pain intensity. On the day of the intervention, after an initial EEG set-up and TENS calibration (see Supplementary Section: Intervention calibration procedure) (Fig. S1), patients underwent an offline recording while in VR. They were instructed to focus on pain every time the “focus on pain” panel appeared in the VR scenario (condition P, Fig. 2c) and to rest while no panel was present (condition R). The recording lasted 6 min and every minute was divided into 30 s of rest and 30 s of focus on pain. At the end of the recording, patients were asked to report on an NPRS their pain intensity while focusing and not focusing on pain. EEG data from the first recording was processed following the same procedure described for healthy with the only difference of using a 1-s ROW with no overlap. This choice was dictated by the higher number of samples for the training of the classifier with respect to healthy, as well as the wider time span from which ROWs are extracted. Extracted EEG features were employed to train an SVM classifier (*C* = 1 and *γ* = 0.01) to distinguish between P and R conditions. SC signal was discarded from the analysis since the two conditions (P and R) did not produce any significant difference in the signal (Fig. S2). During the online phase, patients were again instructed to focus on their pain when the correspondent panel appeared. A majority vote algorithm was here adopted to produce a final classification based on the last three EEG classifications. Given the slower dynamic of this protocol compared to healthy (30 s vs 4 s), we divided each condition (focus vs non focus) into chunks of 6 s. If in this period the classifier detected pain, the intervention was released.

**Table 3:** Temporal characteristics of pain. Q4 and Q7 of the neuropathic pain symptom inventory for each patient

	***Q4: During the past 24 h, your spontaneous pain has been present:***	***Q7: During the past 24 h, how many of these pain attacks have you had:***
**P1**	Permanently	More than 20
**P2**	Permanently	More than 20
**P3**	Between 8 and 12 h	Between 11 and 20
**P4**	Permanently	Between 6 and 10
**P5**	Permanently	More than 20
**P6**	Between 1 and 3 h	Between 1 and 5
**P7**	Permanently	Between 6 and 10
**P8**	Between 1 and 3 h	Between 1 and 5
**P9**	Permanently	Between 1 and 5

### Statistical Analysis

For each of the analyses, the normality was tested using the Shapiro-Wilk tests (the null hypothesis is that the sample comes from a normal distribution). When the null hypothesis was rejected, non-parametric tests were employed. In the intervention validation analysis, the reported NPRS across the four conditions were compared in MATLAB R2021a using a non-parametric Friedman test (Fisher’s post hoc). For patients, the perceived pain intensities (NPRS) during the “focus on pain” and “rest” conditions, as well as the NPSI scores the day before and after the intervention, were compared in MATLAB R2021a using a paired *t*-test. The statistical analysis of the extracted EEG and SC features was performed in Python. Normalized (*z*-scored) feature values for the three conditions (P, NP, R) among healthy subjects were compared with the Friedman one-way repeated measure analysis of variance by ranks, followed by the Siegel-Castellan post hoc test. For patients, features across P and R conditions were compared with a paired *t*-test.

## Results

### Validation of the Multisensory Intervention

The multisensory intervention combined an immersive VR showing a beach scenario and a targeted neural stimulation (Fig. 1a). Analyzing the NPRS across the four conditions (Fig. 1c), the intervention composed of the combination of VR and TENS yielded significantly (Shapiro–Wilk test, *p*-value < 0.05, Friedman test, all *p* < 0.05) lower perceived pain intensity with respect to all the other conditions *(* ${MDN}_{TENS+VR}=6, {Q1}_{TENS+VR}=5, {Q3}_{TENS+VR}=7;$ ${MDN}_{TENS}=7, {Q1}_{TENS}=6, {Q3}_{TENS}=7;$ ${MDN}_{VR}=8, {Q1}_{VR}=7, {Q3}_{VR}=8;$ ${MDN}_{CTRL}=8; {Q1}_{CTRL}=7, {Q3}_{CTRL}=9$*).* Moreover, by evaluating the temporal evolution of reported pain, we found that the VR + TENS condition is associated with the highest slope of decrement ($VR+TENS=-0.023; TENS=-0.005; VR=-0.009; CTRL=-0.004$) (Fig. 1d).

### BCI Offline Classification Results

**Fig. 3:**
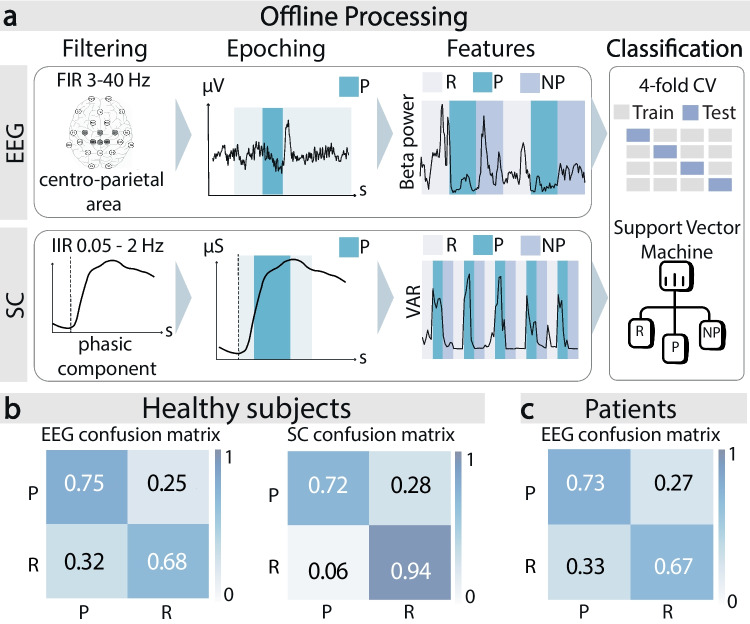
Offline neurophysiological signals processing and results. **a** Offline processing of EEG (upper row) and SC (lower row). EEG signals from six selected channels (Cz, C3, C4, CPz, CP1, CP2) are band-pass filtered (3–40 Hz FIR filter) and 500 ms running observation windows are extracted. Frequency- and entropy-based features are computed (beta power is shown as an example) and fed into a radial kernel SVM. Similarly, the SC signal is filtered, and windows are extracted. Then, amplitude-based features are computed (variance is shown as an example) and fed into a radial kernel SVM. Offline performances are tested through a fourfold cross-validation both for the EEG and SC classifiers. **b** Offline classification results for healthy subjects. EEG and SC raw-normalized confusion matrices for the 2-classes (P vs R) analysis following cross-validation. The average among *N* = 13 subjects is shown for each cell. **c** Offline classification results for patients. EEG raw-normalized confusion matrix for the 2-classes (P vs R) analysis following cross-validation. The average among *N* = 9 patients is shown for each cell

The data collected during the BCI offline phase was processed (filtering, epoching) to extract EEG and SC features (Table 2) training support vector machine (SVM) models decoding pain (Fig. 3a). The offline performances were tested with a fourfold cross-validation which yielded the following results. For healthy subjects, the EEG 2-classes (P vs R) classification model reached 72 ± 3% (mean ± SD) accuracy (i.e., the ratio between the total number of correctly classified samples to the total number of samples) and 75 ± 3% pain recall (i.e., the ratio between the number of correctly classified pain samples to the total number of pain samples) (Fig. 3b). The 3-classes (P vs NP vs R) accuracy was 50 ± 6% (chance level = 33%), while pain recall was 65 ± 7% (Fig. S3). Overall, the SC classifier performed better than EEG (Fig. 3b). Classification accuracy was 83 ± 5% for the 2-classes analysis and 57 ± 5% for the 3-classes analysis. For what concerns pain recall, it reached 72 ± 11% for the 2-classes analysis (Fig. 3b) and 71 ± 9% for the three classes analysis (Fig. S3).

For patients, the reported NPRS was significantly higher in P condition ${NPSI}_{P}=4.75\pm 2.82$ (mean ± SD) compared to R condition ${NPSI}_{R}=1.62\pm 1.60$ (Shapiro–Wilk test, *p*-value > 0.05; one-tail paired *t*-test, *p*-value = 0.014, Cohen’s effect size = 1.29, statistical power = 0.95) (Fig. 2c). Offline, the SVM classifier trained on EEG data reached 70 ± 5% accuracy and 73 ± 6% pain recall (Fig. 3c).

### Neurophysiological Features Analysis

**Fig. 4:**
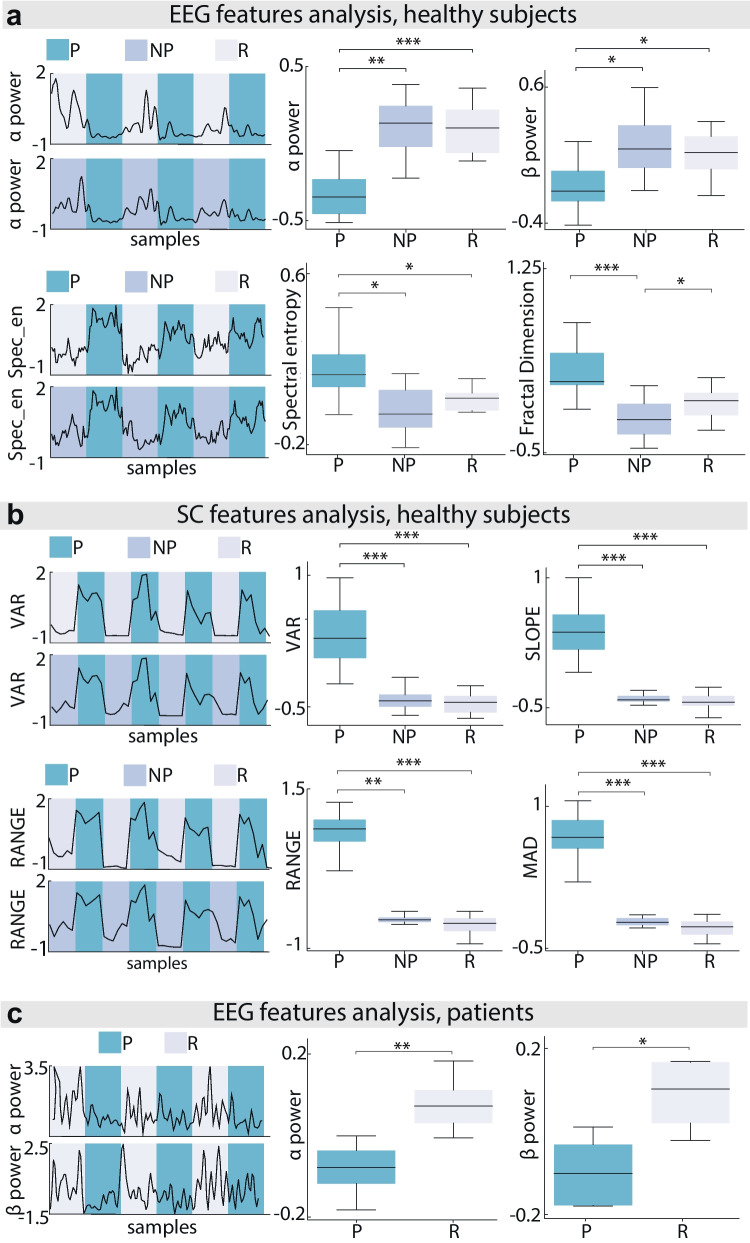
Features statistical analysis. EEG (**a**) and SC (**b**) features analysis for healthy patients. On the left, a graphical representation of features values for P (blue) vs R (gray) and P vs NP (purple) comparisons for one illustrative subject. On the right, examples of features significantly different in P with respect to NP and/or R are shown. Each boxplot contains data from *N* = 13 subjects. **c** EEG features analysis for patients. On the left, is a graphical representation of features values for P vs R comparison for one illustrative patient. On the right, the features significantly different in P with respect to R are shown. Each boxplot contains data from *N* = 9 subjects (* *p* < 0.05, ** *p* < 0.01, *** *p* < 0.001)

For healthy subjects, some EEG and SC features were not normally distributed (Shapiro–Wilk test, *p* < 0.05). Therefore, to keep the analysis consistent, non-parametric tests were used for all features. EEG alpha power was significantly lower in P with respect to NP (*p* < 0.001 Friedmann) and R (*p* < 0.01) conditions, while no statistical difference was found for the NP vs R comparison (Fig. 4a, for effect sizes, refer to Table S2). Similarly, beta power had a significant decrease in P compared to NP (*p* < 0.05) and R (*p* < 0.05). Again, no statistical difference was found for the NP vs R comparison conditions (Fig. 4a and Table S2). The same trend was followed as well by overall signal power (Table S2). None of the other frequency-based features yielded significant results both for P vs NP and for P vs R comparisons at the same time (Table S2). For what concerns entropy features, condition P had a significant increase in spectral entropy compared to R (*p* < 0.05) and to NP (*p* < 0.05) (Fig. 4a and Table S2). Similarly, fractal dimension was found to increase in P with respect to NP (*p* < 0.001) (Fig. 4a and Table S2), while sample entropy increased in P with respect to R (*p* < 0.05) (Table S2). Finally, RMS significantly decreased in P with respect to R (*p* < 0.05) and NP (*p* < 0.05) conditions (Table S2). Almost all the SC features were statistically different in the condition of P with respect to NP and R (Table S3). Graphical examples of variance (VAR), slope, range, and mean absolute deviation (MAD) are reported in Fig. 4b. For patients, the Shapiro–Wilk test null hypothesis was not rejected for any of the features; therefore, parametric analysis was performed. As for healthy subjects, alpha, beta, and 0–40 Hz power for patients were found to decrease in the painful condition with respect to the resting condition (Fig. 4c and Table S4) (two-tails paired *t*-test, *p*-value < 0.05), thus highlighting an overall decrease of the signal power in these bands as a possible signature of increased perceived pain (for effect sizes, refer to Table S4). The same behavior was found for RMS, which decreased in P also for patients (Table S4). The other frequency-based features and entropy features did not yield significant results (Table S4).

### BCI Online Performance

**Fig. 5:**
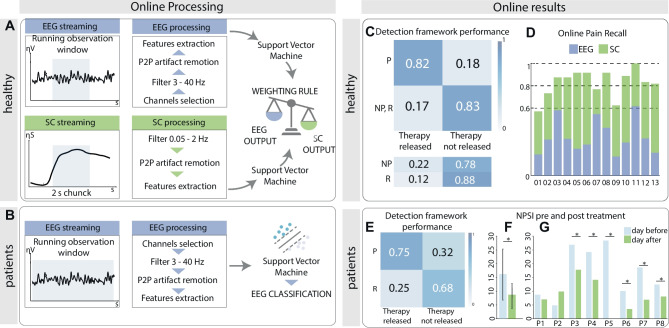
Online BCI outline and results. **A** Online processing for healthy subjects. 500 ms EEG chunk and 2 s SC chunk are processed to produce a classification every 500 ms each. EEG and SC classifications are then weighted and merged to produce a final classification. **B** Online processing for patients. EEG signal is streamed and processed to produce a classification every second. **C** Detection framework performances for healthy subjects. In the upper part is a raw-normalized confusion matrix for online pain detection. In the lower part, in-depth analysis of triggered therapy stimulation following NP and R stimulations. **D** EEG and SC contributions to correct pain classification for each healthy subject. **E** Detection framework performances for patients. Column-normalized confusion matrix for online pain detection. **F** NPSI scores on the day of the therapy (light blue) and the day after the therapy (green) among *N* = 8 patients (mean $\pm$ SD). (* *p* < 0.05). **G** NPSI scores on the day of the therapy (light blue) and the day after the therapy (green) for each of the patients. NPSI score decreases of over 30% were marked as clinically relevant

For the online implementation, we purposely developed processing pipelines shaped for healthy subjects (Fig. 5a) and patients (Fig. 5b) to detect pain in real time. Healthy subjects’ online pain recall was 82 ± 3% (mean ± SD); hence, 82 ± 3% of the time that the subjects received a painful stimulation (P) the BCI the intervention combining VR and TENS was released. On the other hand, when the subjects received non-painful (NP) stimulation, the intervention was released only 22 ± 4% of the times, and when subjects received no stimulation, the intervention was released 12 ± 3% of the times (Fig. 5c). To reveal which neurophysiological recording was most important for the pain classifications, it is interesting to analyze the performance of EEG and SC classifiers separately. We, therefore, quantified the classifiers’ contribution to correct pain classifications and found that the average EEG contribution was 42 ± 16% (mean ± SD), while SC contribution was 58 ± 16% (Fig. 5d). This result shows that both classifiers are actively contributing to improving the system performances. For patients, the designed online pipeline allowed the BCI to reach 75% ± 7% precision (i.e., the ratio between the total number of correctly classified pain to the total number of pain classifications); hence, most of the times that the intervention was released patients were perceiving higher pain (Fig. 5e). Results in terms of pain recall were lower, reaching 65 ± 10% among subjects. For what concerns pain assessment, the NPSI score before the intervention was 16.64 ± 8.91 (mean ± SD), while the day after the intervention the NPSI was significantly lower (8.35 ± 5.58, 50% decrease; Shapiro–Wilk test, *p*-value > 0.05; one-tail paired *t*-test; *p* = 0.022, Cohen’s effect size = 1.05, statistical power = 0.85) (Fig. 5f). Analyzing the results patient per patient (Fig. 5g), we noticed a substantial pain decrease (higher than 50%) [54–56] in P5, P6, and P7 (${NPSI}_{P5}^{pre}=28.17$, ${NPSI}_{P5}^{post}=0$; ${NPSI}_{P6}^{pre}=10.00$, ${NPSI}_{P6}^{post}=3.50$; ${NPSI}_{P7}^{pre}=18.50$, ${NPSI}_{P7}^{post}=6.83$) and a moderate [54–56] pain decrease (higher than 30%) in P3, P4, and P8 (${NPSI}_{P3}^{pre}=26.67$, ${NPSI}_{P3}^{post}=17.67$; ${NPSI}_{P4}^{pre}=24.00,$ ${NPSI}_{P4}^{post}=14.00$; ${NPSI}_{P8}^{pre}=12.33$, ${NPSI}_{P8}^{post}=8.00$).

## Discussion

This study developed and validated a BCI for neuropathic pain. Overall, our results demonstrate the feasibility of a real-time pain detection system to provide a holistic intervention leading to pain relief. There has recently been a growing interest in the development of closed-loop therapies to promote personalized treatments. Sun et al. [44] proposed a BCI able to detect pain in mice brains and provide real-time therapy delivery. To the authors’ knowledge, however, our system is the first example of a therapeutic BCI detecting pain through EEG and SC in humans.

Our BCI successfully detected induced pain in real time reaching over 82% pain accuracy. The purposely designed probabilistic approach for the classification allowed a functional integration of EEG and SC. Indeed, even though SC’s contribution to correct classifications was higher, both classifiers actively contributed to enhancing classification performances. Moreover, it would not have been possible to rely only on SC for our classification because of the low informative content of this signal, which could produce an identical peak for any salient stimulus (i.e., threat). Even though the detection framework showed promising results for patients as well (75 ± 7% precision), BCI online performances were higher for healthy subjects compared to patients. This is likely linked to how patients had to focus on their pain, rather than receiving experimentally induced pain as in healthy. The absence of a transient and fast stimulus prevented a clear signature of pain in SC, which was therefore discarded in the patients’ processing pipeline. As a result, a functional and effective integration between the two neurophysiological signals (EEG and SC) was possible in healthy subjects only.

In the EEG features analysis, our results showed a common trend of decreased alpha power in the centro-parietal area following a painful electrical stimulation. This is consistent with the majority of pain-related studies, which report a decrease of the alpha band as a biomarker of pain perception after experimentally induced pain [22–24, 26]. However, we found also the same trend for the beta band, which has been shown in previous literature to have a more variable behavior, hence having a role still highly debated in induced pain paradigms [22, 23, 27, 57, 58]. No significant results were found for the gamma band, even though in literature [59] there is an overall agreement over increased gamma power correlated pain induction. Gamma oscillations are thought to encode the top-down subject-driven cognitive components of pain [60]. In this view, the employed VR scenario may have directly interacted with subjects’ cognitive pain processing, leading to high response variability reflected by the absence of gamma significance.

Despite the different pain protocols employed for healthy subjects and patients, common trends emerged from the offline features analysis. Indeed, also for patients and similar to previous studies [30, 31, 61], both alpha and beta power decreased while experiencing higher pain compared to the rest condition. Nevertheless, other studies reported contradictory results [29] highlighting instead an increase of beta as a possible biomarker of neuropathic pain. These similarities in results between healthy subjects and patients highlight the potential of the BCI in detecting a painful state regardless of its nature. Indeed, even though we specifically asked the patients to focus on their pain to increase its perception, this did not seem to drastically affect the pain signatures and therefore did not affect the efficacy of the BCI in releasing the therapy at the right moment. EEG features analysis conducted on healthy subjects also showed significant increase of entropy features as a possible biomarker of pain. This means that the signal during rest is overall more repetitive compared to the pain condition, which instead generates uncertainty and decreases the predictability of the signal. For what concerns SC, the peak response in the signal during the P condition yielded significant results not only in comparison to the R condition, but also to the NP condition, meaning that skin conductance features are encoding pain-induced arousal rather than arousal caused by any electrical stimulation. The fact that all of the chosen features were significantly different in P condition could also explain the higher performance of this signal in both the online and offline classifications. The proposed SC signal processing pipeline is different from the majority of studies, which use wider observation windows because of its slow dynamics. Nevertheless, our methods are justified by the necessity of extracting pain-related content with the lowest possible delay. This is functional within the schema of a real-time neurophysiological features system detection. The final objective of a real-time pain detection system influenced the choice of EEG processing pipeline as well. Indeed, due to the time constraints of the online system, we avoided implementing an online independent component analysis for artifact removal, but rather used an immediate peak-to-peak artifact. Moreover, envisioning a home-use scenario of the device, it is of utmost importance to improve the usability, portability, and simplicity of the system. This ideally means having a headset with the lowest possible number of electrodes. For this reason, we focused only on 6 channels of the centro-parietal area. This prevented the usage of complex filtering techniques like source localization and Laplacian filtering, which could have significantly improved the decoder performances. These were the main reasons why the offline pain classification results were slightly inferior to some of the studies in the literature, which instead adopted some of those techniques [45, 62–64].

Moving towards the effectiveness of the intervention, we proposed a novel therapeutic approach based on the combination of TENS and VR. Before implementing the real-time BCI system, the efficacy of the proposed intervention was demonstrated on healthy subjects by showing that the combination of TENS and VR provided a significant decrease in pain with respect to the unimodal approaches. It is worth noticing that contrary to the VR condition, the unimodal TENS condition led to a significant improvement with respect to the control. However, when this technology is coupled with VR, pain perception decreases even further. This result highlights the VR potential as a complementary element to conventional pain therapy rather than as a stand-alone therapeutic solution.

When the intervention was implemented in the BCI framework and tested on neuropathic patients, there was a significant decrease in pain (50% NPSI score decrease) the day after the session, showing the potential of this novel, safe, and non-invasive intervention. Due to the absence of a control condition, we can only speculate on the reasons behind the effectiveness of the proposed intervention. Given the importance of attention modulation in our experimental settings, the VR scenario likely played a role in patients’ pain decrease. However, while VR has shown also in other studies to be able to decrease pain through attentional mechanisms, it is known to have an analgesic effect while, or at least immediately after that, the patient is immersed in the VR environment [42]. Therefore, since we collected data on pain decrease 24 h after the intervention, the distraction from pain cannot entirely explain the observed NPSI reduction.

Finally, the benefits of this intervention could be further expanded to beneficially impact altered body representations. Indeed, recent research has shown that patients with diabetic neuropathy have a cortical reorganization similar to the one found in amputees [65]. While there is no universal consensus on the exact mechanisms behind these plastic changes, one of the potential explanations relies on the incongruencies between the visual feedback and the motor intention which create sensory-motor conflicts [66]. Even though several approaches have been proposed to restore correct communication between these systems (i.e., Mirror Box [67], Phantom Motor Execution [68]), the benefits of using plasticity-based interventions in neuropathic pain populations are still to be explored. It is important to notice that the multisensory stimulation has to be synchronous, meaning that the sensory inputs have to be temporally matching. This temporal rule is fundamental both to manipulate body ownership [69, 70] and to provide benefits for altered body representations [71, 72] and chronic pain [73]. Therefore, with the combination of VR and TENS, we aimed at providing synchronous visuo-tactile feedback from the extremities to reduce sensory-motor conflicts in patients affected by chronic neuropathic pain. However, to confirm the potential cortical changes and similarly to what has been done for invasive stimulation [74], further studies collecting fMRI data would be necessary.

### Limitations and Future Perspectives

To the best of the authors’ knowledge, this study has shown for the first time the possibility of detecting pain and delivering a purposely designed intervention in real time for neuropathic patients. However, it presents some limitations. Firstly, the proposed BCI relies on two different communication protocols for EEG and SC signals (LSL and OSC respectively). The use of two different protocols does not allow the precise synchronization of the streamed signal and therefore requires two different processing pipelines, discarding the possibility of building a unique classifier for both signals. On the other hand, to limit the potential negative outcomes of this failed integration, a versatile probabilistic approach merging weighted information from SC and EEG classifiers was built and yielded successful results.

Moving towards the efficacy of the intervention, it is necessary to stress that, despite noticing a significant pain reduction 1 day after the BCI testing, we cannot completely rule out a placebo effect nor indicate which intervention between TENS and VR had the higher effect. Indeed, despite having validated the intervention on healthy subjects using proper control conditions (i.e., only VR and only TENS), these have to be performed in the future with patients. In this proof-of-concept study for time purposes, we tested the complete intervention only.

Furthermore, the different protocol employed for healthy subjects and patients is another important limitation. While in healthy subjects we elicited a transient arousal with a painful electrical stimulation, for patients, we did not induce pain but rather modulated their attention to pain. These changes explain why SC recording in patients did not yield significant results. As of today, the BCI structure developed for healthy would perfectly fit neuropathic patients with sharp, episodical attacks of pain, or patients who have painful/pain-free phases. This would allow the integration of SC decoding for neuropathic patients as well and therefore more coherent and consistent results. However, to test the performances of the system with such patients, it would be necessary to monitor, for instance in a home-use scenario, electrodermal and brain-related activity over several hours or days and train the classifier on the features extracted from these recordings. Since we performed the BCI testing onsite at the laboratory, we validated the BCI by slightly changing some of its characteristics to fit the patients’ pain profile. Despite possible spontaneous pain attacks, we chose to target the attentional component of pain to avoid daunting sessions waiting for a pain attack to occur. Nevertheless, we want to highlight that our final aim is not to propose an intervention where the patients must focus on their pain (hence increasing it, which could be problematic from a behavioral-therapeutic point of view). Instead, this was a methodological choice that could simulate their pain attacks, to make the experiments more uniform as possible among patients and temporally feasible. Indeed, given that the patients reported to have several pain fluctuations, we envision the possibility of using our system in a home scenario, where the BCI would then detect pain attacks rather than focusing on pain. In this view, we need to stress that the current setting of this proof-of-concept BCI is not ready for real-life clinical contexts and future studies with a longitudinal design should investigate the feasibility and potential of training and testing the BCI on spontaneous pain attacks.

Continuing with the future implications, while the current BCI cannot discriminate between various components of pain, this represents an interesting perspective towards the personalization of the intervention. More specifically, future studies with a high sample size could aim at distinguishing biomarkers for the sensory and attentional components of pain. For example, if the BCI would be able to detect specifically the attentional component of pain in hypochondriac patients (i.e., where the sensory component is absent), it could fine-tune the intervention targeting that specific component, which in our case would be the VR modulation*.*

Finally, for future use, our system could be exploited as an objective automatic detection system for pain for several real-time applications. The intervention we designed is indeed specific for neuropathic patients. Nevertheless, VR and TENS output could be substituted with other treatments for different etiological conditions of pain.

## Supplementary Information

Below is the link to the electronic supplementary material.

Supplementary file1 (DOCX 538 kb)

## Data Availability

The dataset generated during the study, together with the processing codes employed during this research, is available from the corresponding author on reasonable requests.
