# Supplementary material for: Brain-Computer Interface to Deliver Individualized Multisensory Intervention for Neuropathic Pain
Source: Neurotherapeutics. 2023 Jul 5;20(5):1316–29. doi: 10.1007/s13311-023-01396-y (PMC10480109; doi:10.1007/s13311-023-01396-y)
Supplement: Supplementary file 1 — Supplementary file1 (DOCX 538 kb) [file 13311_2023_1396_MOESM1_ESM.docx]

**Supplementary Information**

**Brain-computer interface to deliver individualized multisensory intervention for neuropathic pain**

**List of Supplementary Materials:**

**Pain calibration procedure**

**Intervention calibration procedurea**

**Probabilistic approach**

**Fig. S1. Electrical calibration results**

**Fig. S2. SC features analysis**

**Fig. S3. 3-classes offline classifiers performance**

**Table S1: EEG and SC features extended**

**Table S2. Results of the EEG statistical analysis for healthy subjects**

**Table S3. Results of the SC statistical analysis for healthy subjects**

**Table S4. Results of the EEG statistical analysis for patients**

**Table S5. Neuropathic pain symptoms inventory (NPSI) the day before the intervention for all patients.**

**Pain calibration procedure**

The pain calibration was performed both for the intervention validation protocol (5 subjects) and for the BCI protocol (13 subjects) to find personalized stimulation parameters for P and NP conditions. The stimuli duration was 100 ms for the intervention validation protocol and 4 s for the BCI protocol. The electrodes were placed on the dorsal side of the foot. The calibration consisted of two phases:

1. Subjects received 50 Hz stimuli of fixed pulse width (100 µs) and increasing pulse amplitude and were asked to report a 5/10 intensity sensation
2. Subjects received 50 Hz stimuli of fixed amplitude (the one just saved) and increasing pulse width and were asked to mark a non-painful sensation (NP, 2/10 intensity) and the maximum tolerable pain (P, 9/10 intensity). This step was repeated three times. Finally, the means of the pulse widths for P and NP conditions were saved.

Once the calibration was completed subjects were asked to fill a topographic map by inserting the location where they felt the stimulation (Fig. S1).

**Intervention calibration procedure**

We performed the intervention calibration to find personalized TENS parameters for tibial and peroneal nerves. While for healthy subjects the TENS intervention was provided only on one foot, for patients we employed up to 4 channels (two nerves for foot) depending on the location of the reported pain. For each of the nerves, the intervention calibration consisted of two phases.

1. Electrodes were placed in proximity of the target nerve. Subjects received 2 s stimuli (50 Hz) of fixed pulse width (100 µs) and increasing amplitude and were asked to describe the location of a 4/10 intensity perceived sensation. The electrodes placement was changed until it ensured the spreading of the sensation alongside the desired nerve to cover the area where the pain was felt/induced.
2. Subjects received 2 s stimuli (50 Hz) of fixed amplitude (the one just saved) and increasing pulse width. They were asked to mark the first perceived sensation (1/10 intensity) and a sensation intense and comfortable enough to be repeatedly received. These values corresponded to the minimum and the maximum of the modulated wave respectively (Fig. 1A).

Again, subjects filled a topographic map showing where they felt the stimulation (Fig. S1).

**Probabilistic approach**

In the online pipeline, EEG and SC classifiers acted independently to produce a classification every 500 ms each. A 2-classes (P vs R) probabilistic approach was employed to merge the information coming from both classifiers. According to this approach, the probability that the person was feeling pain was updated every time a new classification (from EEG or SC) was produced. At a specific time point, the probability for the subject to experience pain was:

$$p\left( y_{t} \right)=\alpha\cdot p\left( y_{t-1} \right)+\left( 1-\alpha\right)\cdot p_{eeg,sc}\left( y_{t} | x_{t} \right)\cdot w_{eeg,sc}$$

Where:

1. α is a parameter whose value can go from 0 to 1, depending on how much importance is given to the past classifications. In our system, α was chosen to be 0.5;
2. $p\left( y_{t-1} \right)$ corresponds to the probability of the person feeling pain at the previous time stamp. Note that $p\left( y_{t-1} \right)$ is initialized to $p\left( y_{0} \right)=0.5$;
3. $p_{eeg,sc}\left( y_{t} | x_{t} \right)$ corresponds to the output of the last classification (in terms of pain probability), which could come both from EEG or SC models;
4. $w_{eeg,sc}$ is a weight that depends on how well SC and EEG have performed offline. It weights the impact that the classification just received $p_{eeg,sc}\left( y_{t} | x_{t} \right)$ should have on the final probability $p\left( y_{t} \right)$. Here follows its definition

$$w_{eeg,sc}=\frac{k_{eeg,sc}\cdot p_{eeg,sc}\left( y_{t} | x_{t} \right)+\left( 1-k_{eeg,sc} \right)\cdot p\left( y_{t-1} \right)}{p_{eeg,sc}\left( y_{t} | x_{t} \right)}$$

The parameter $k_{eeg,sc}$ goes from 0 to 1 and regulates the percentage of the highest possible change from $p\left( y_{t-1} \right)$ to $p\left( y_{t} \right)$ depending on how much we want to rely on EEG or SC classification.
If $k_{eeg,sc}=0$ then $w_{eeg,sc}=0$ and the new classification $p_{eeg,sc}\left( y_{t} | x_{t} \right)$ does not influence at all the current probability $p\left( y_{t} \right)$. On the other hand, if $k_{eeg,sc}=1$, then $w_{eeg,sc}=1$ and the new classification guarantees the highest possible increase of $p\left( y_{t} \right)$. If $k_{eeg,sc}=0.5$ the new classification guarantees half of the highest possible increase of $p\left( y_{t} \right)$. The value of $k_{eeg,sc}$ was chosen before the online session, depending on the offline accuracy of EEG and SC 2-classes, according to the following Table.

|  | $acc_{sc}\geq acc_{eeg}$ | $acc_{sc}<acc_{eeg}$ |
| --- | --- | --- |
| $\vert acc_{sc}-acc_{eeg}\vert\geq0.3$ | $k_{sc}=1, k_{eeg}=0.5$ | $k_{sc}=0.5, k_{eeg}=1$ |
| $0.1\leq\vert acc_{sc}-acc_{eeg}\vert<0.3$ | $k_{sc}=1, k_{eeg}=0.75$ | $k_{sc}=0.75, k_{eeg}=1$ |
| $\vert acc_{sc}-acc_{eeg}\vert<0.1$ | $k_{sc}=1, k_{eeg}=1$ | $k_{sc}=1, k_{eeg}=1$ |

$\boldsymbol{k}_{\boldsymbol{eeg,sc}}$ **values**

**^
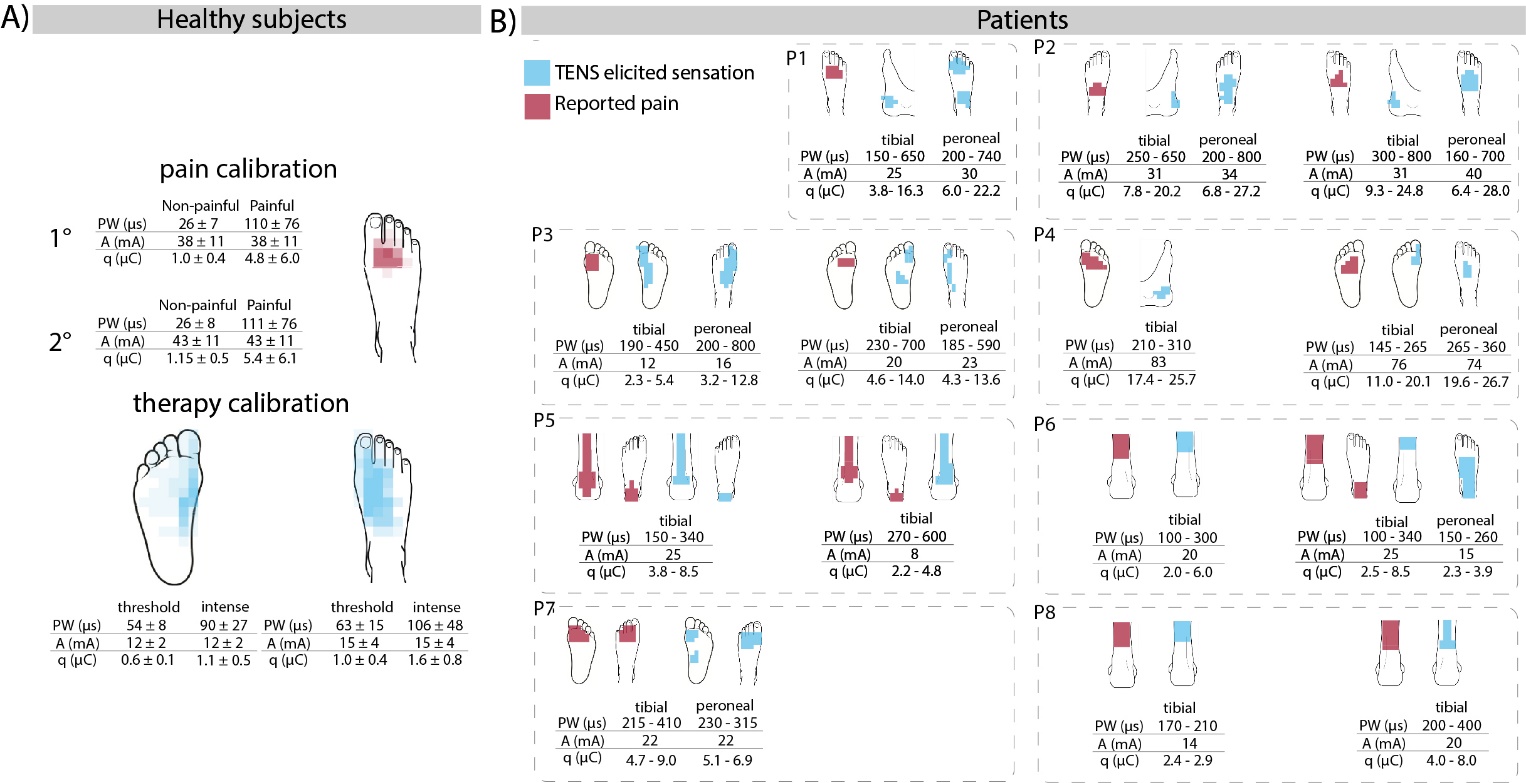
^Fig. S1. Electrical calibration results.** A) Pain and TENS intervention calibration for 13 healthy subjects who participated in the BCI study. ­ In the upper part, TENS parameters for P and NP conditions for the first and second calibration (mean $\pm$ SD). In the lower part, TENS parameters for the tibial (plantar) and peroneal (dorsal) nerves (mean $\pm$ SD). The two pulse width values for each nerve correspond to the minimum (threshold) and the maximum (intense) of the modulated wave respectively. The darker the color of the area, the higher the number of subjects who reported pain in that area. B) Patients intervention calibration. For each patient, the reported painful locations are shown in red, while the map of TENS elicited sensation is shown in blue. In the tables, TENS intervention parameters for the tibial and peroneal nerves are reported for each foot.


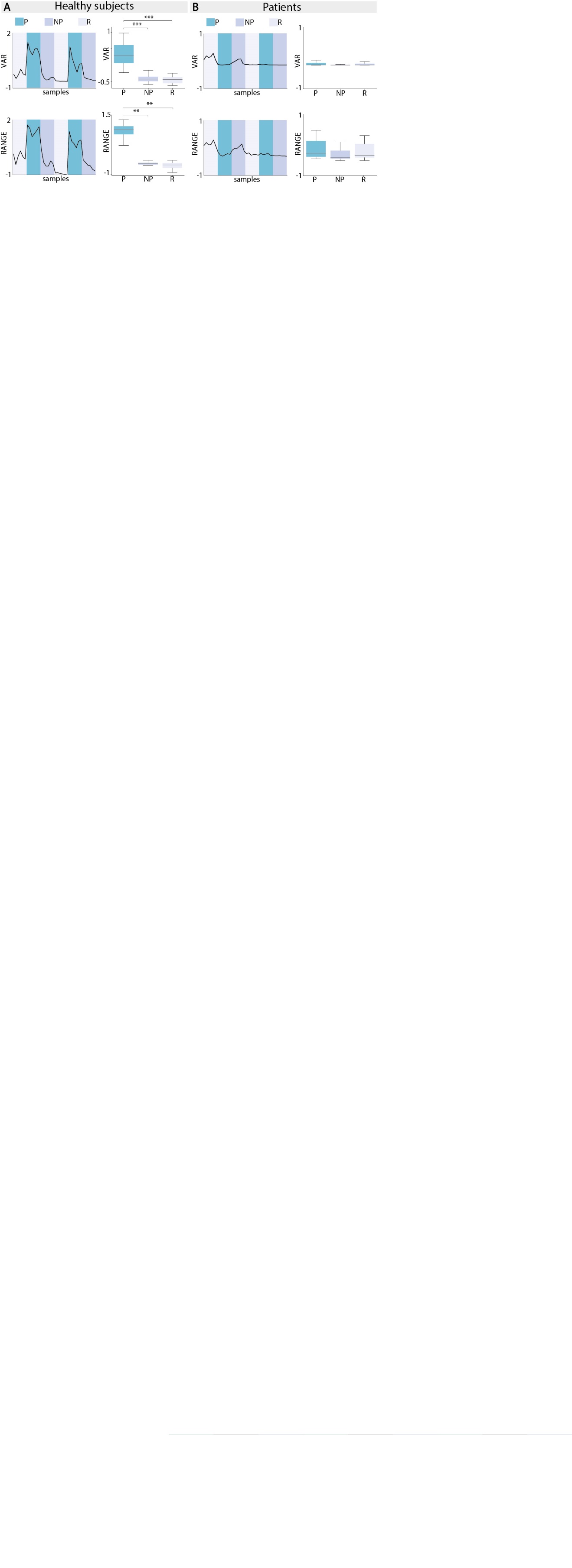


**Fig. S2. SC features analysis.** A) SC features analysis for healthy subjects. On the left, a graphical representation of features values for P (blue) vs R (grey) and P vs NP (purple) comparisons for one illustrative subject. On the right, examples of features significantly different in P with respect to NP and R are shown. Each boxplot contains data from N=13 subjects. B) SC features analysis for one illustrative patient. On the left, is a graphical representation of features values for P (blue) vs R (grey) comparisons. On the right, boxplots of VAR and RANGE features are shown. (* p < 0.05, ** p < 0.01, *** p < 0.001).


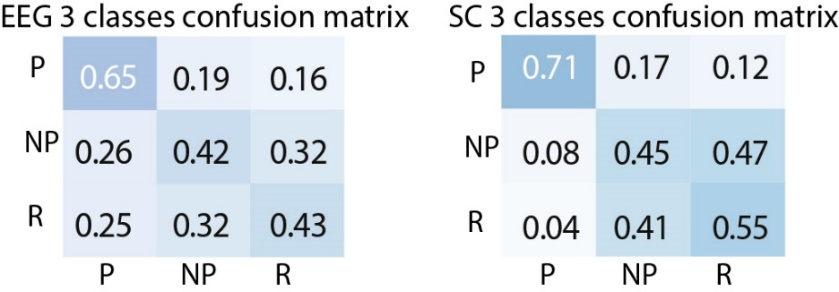


**Fig. S3. 3-classes offline classifiers performance.** Offline 3-classes classification results for healthy subjects. EEG (on the left) and SC (on the right) raw-normalized confusion matrix for the 3-classes (P vs NP vs R) analysis following cross-validation. The average among N=13 subjects is shown for each cell.

| $Theta Power$ $=\int_{4 Hz}^{8 Hz} S\left( f \right)$  $S\left( f \right)=\frac{1}{N}\left\vert X\left( 2 \pi f \right)^{2} \right\vert$*; X*$\left( w \right)=\sum_{i=1}^{N-1} {\left\vert x_{i}^{2} \right\vert e}^{-j 2 \pi n}$ | $Mean$ |
| --- | --- |
| $Alpha Power$ $=\int_{8 Hz}^{13 Hz} S\left( f \right)$ | $Max$ |
| $Beta Power$ $=\int_{13 Hz}^{30 Hz} S\left( f \right)$ | $Median$ |
| $Gamma Power$ $=\int_{30 Hz}^{40 Hz} S\left( f \right)$ | $Standard deviation$ |
| $Power 0-40 Hz=\int_{0 Hz}^{40 Hz} S\left( f \right)$ | $Variance$ |
| $Peak frequency=\arg\max_{f} S\left( f \right)$ | $IQR=3^{th}interquantile-1^{st}interquantile$ |
| $Root mean square$ *=* $\sqrt{\frac{1}{N}\sum_{i=1}^{N} x_{i}^{2}}$ | $Root mean square$ *=* $\sqrt{\frac{1}{N}\sum_{i=1}^{N} x_{i}^{2}}$ |
| $Sample entropy= -log\left( \frac{A}{B} \right)$  $A: \# of vectors having$ $d\left[ x_{m+1}\left( i \right),x_{m+1}\left( j \right) \right]<r \left( i\neq j \right),i,j=1,N$*;*  $B:\# of vectors having$ $d\left[ x_{m}\left( i \right),x_{m}\left( j \right) \right]<r \left( i\neq j \right),i,j=1,N$*;* $m=2$*;* $d is the Chebyshev distance$ | $Range=Max \left( x \right)-Min \left( x \right)$ |
| $Spectral entropy=-\sum_{1}^{N} P\left( f \right)\log_{2} \left( P\left( f \right) \right)$  $P\left( f \right)=\frac{S\left( f \right)}{\sum_{i} S_{i}\left( f \right)}$*;*$S\left( f \right) Power Spectral Density$ | $Slope=Slope of the best fitting linear function$ |
| $Higuchi^{'}s Fractal Dimension==Slope of the best fitting linear function through (\log\frac{1}{k} ,\log\frac{L}{k})\}$ $L\left( k \right)=\frac{1}{k} \sum_{m=1}^{k} L_{m\left( k \right)} ;$ $L_{m}\left( k \right)=\frac{N-1}{\left\lfloor\frac{N-m}{k} \right\rfloor k^{2}}\sum_{i=1}^{\left\lfloor\frac{N-m}{k} \right\rfloor} \vert x_{m+ik}-x_{m+i-1k}\vert$  $k=1,k_{max}$*;* $k_{max}=10$ | $Mean Absolute Deviation=\frac{1}{N}\sum_{i=1}^{N} \vert x_{i}-\mu\vert$ |
|  | $Area Under Curve= \frac{1}{2}\sum_{i=1}^{N} {(x}_{i+1}-x_{i})$ |

**Table S1: EEG (left) and SC (right) features extended**

|  | *theta power* | *alpha power* | *beta power* | *gamma power* | *peak frequency* | *power*  *3-40 Hz* | *sample entropy* | *spectral entropy* | *fractal dimension* | *rms* |
| --- | --- | --- | --- | --- | --- | --- | --- | --- | --- | --- |
| *p vs r* | *ns* | *↓↓↓*  *ES=2.71* | *↓*  *ES=0.93* | *ns* | *ns* | *↓*  *ES=1.70* | *↑*  *ES=1.23* | *↑*  *ES=1.35* | *ns* | *↓*  *ES=1.57* |
| *p vs np* | *ns* | *↓↓*  *ES=2.62* | *↓*  *ES=1.07* | *ns* | *↓*  *ES=0.87* | *↓*  *ES=1.61* | *ns* | *↑*  *ES=1.53* | *↑↑↑*  *ES=1.57* | *↓*  *ES=1.61* |
| *np vs r* | *ns* | *ns* | *ns* | *ns* | *ns* | *ns* | *ns* | *ns* | *↓*  *ES=0.66* | *ns* |

**Table S2. Results of the EEG statistical analysis for healthy subjects.** N=13 subjects. Upward arrows indicate that the distribution of that specific feature significantly increases for the first condition with respect to the second. Downward arrows indicate the opposite. Friedman test (↑↑↑: p <= 1.00e-03; ↑↑: p <= 1.00e-02; ↑: p <= 0.05; ns= non-significant). ES: Cohen’s effect size.

|  | *mean* | *slope* | *rms* | *mad* | *auc* | *range* | *iqr* | *std* | *var* | *max* | *median* |
| --- | --- | --- | --- | --- | --- | --- | --- | --- | --- | --- | --- |
| *p vs r* | *↓↓*  *ES=2.24* | *↑↑↑*  *ES=3.40* | *↑↑↑*  *ES=5.33* | *↑↑↑*  *ES=5.00* | *↓↓*  *ES=2.20* | *↑↑↑*  *ES=5.27* | *↑↑↑*  *ES=5.35* | *↑↑↑*  *ES=5.25* | *↑↑↑*  *ES=-2.84* | *ns* | *↓↓*  *ES=1.93* |
| *p vs np* | *↓↓↓*  *ES=2.26* | *↑↑↑*  *ES=3.32* | *↑↑↑*  *ES=4.56* | *↑↑↑*  *ES=4.14* | *↓↓↓*  *ES=2.23* | *↑↑*  *ES=4.60* | *↑↑*  *ES=4.35* | *↑↑↑*  *ES=4.47* | *↑↑↑*  *ES=-2.70* | *ns* | *↓↓↓*  *ES=1.95* |
| *np vs r* | *ns* | *ns* | *ns* | *ns* | *ns* | *ns* | *ns* | *ns* | *ns* | *ns* | *ns* |

**Table S3. Results of the SC statistical analysis for healthy subjects.** N=13 subjects. Upward arrows indicate that the distribution of that specific feature significantly increases for the first condition with respect to the second. Downward arrows indicate the opposite. Friedman test (↑↑↑: p <= 1.00e-03; ↑↑: p <= 1.00e-02; ↑: p <= 0.05; ns= non-significant). ES: Cohen’s effect size.

|  | *theta power* | *alpha power* | *beta power* | *gamma power* | *peak frequency* | *power*  *3-40 Hz* | *sample entropy* | *spectral entropy* | *fractal dimension* | *rms* |
| --- | --- | --- | --- | --- | --- | --- | --- | --- | --- | --- |
| *p vs r* | *ns* | *↓↓*  *ES=1.69* | *↓*  *ES=2.17* | *ns* | *ns* | *↓*  *ES=1.92* | *ns* | *ns* | *ns* | *↓*  *ES=2.02* |

**Table S4. Results of the EEG statistical analysis for patients.** N=9 patients. Upward arrows indicate that the distribution of that specific feature significantly increases for the first condition with respect to the second. Downward arrows indicate the opposite. Paired t-test (↑↑↑: p <= 1.00e-03; ↑↑: p <= 1.00e-02; ↑: p <= 0.05; ns= non-significant). ES: Cohen’s effect size.

The reported NPSI for each patients the day before the experiment is reported in Table S5. The NPSI score (0-50) was calculated summing the five different pain subscales - (1) burning (superficial) spontaneous pain ($Q1)$; (2) pressing (deep) spontaneous pain ($\frac{Q2+Q3}{2})$; (3) paroxysmal pain ($\frac{Q5+Q6}{2})$; (4) evoked pain ($\frac{Q8+Q9+Q10}{3})$; (5) paresthesia/dysesthesia $\left( \frac{Q11+Q12}{2} \right)$- taken from [1]*.*

|  | ***Q1*** | ***Q2*** | ***Q3*** | ***Q4*** | ***Q5*** | ***Q6*** | ***Q7*** | ***Q8*** | ***Q9*** | ***Q10*** | ***Q11*** | ***Q12*** |  |
| --- | --- | --- | --- | --- | --- | --- | --- | --- | --- | --- | --- | --- | --- |
| ***P1*** | *3* | *3* | *2* | *Permanently* | *1* | *0* | *More than 20* | *2* | *0* | *0* | *1* | *3* |  |
| ***P2*** | *1* | *0* | *3* | *Permanenetly* | *0* | *0* | *More than 20* | *1* | *1* | *2* | *2* | *0* |  |
| ***P3*** | *5* | *7* | *8* | *Between 8 and 12 hours* | *8* | *8* | *Between 11 and 20* | *0* | *0* | *5* | *5* | *4* |  |
| ***P4*** | *7* | *7* | *3* | *Permanently* | *5* | *5* | *Between 6 and 10* | *0* | *0* | *0* | *7* | *7* |  |
| ***P5*** | *7* | *2* | *4* | *Permanently* | *5* | *2* | *More than 20* | *5* | *7* | *8* | *8* | *8* |  |
| ***P6*** | *4* | *1* | *2* | *Between 1 and 3 hours* | *0* | *1* | *Between 1 and 5* | *0* | *0* | *3* | *2* | *4* |  |
| ***P7*** | *3* | *7* | *6* | *Permanently* | *0* | *0* | *Between 6 and 10* | *2* | *5* | *5* | *6* | *4* |  |
| ***P8*** | *7* | *0* | *0* | *Between 1 and 3 hours* | *0* | *0* | *Between 1 and 5* | *0* | *7* | *0* | *6* | *0* |  |
| ***P9*** | *3* | *10* | *10* | *Permanently* | *3* | *3* | *Between 1 and 5* | *9* | *5* | *0* | *0* | *5* |  |

**Table S5. Neuropathic pain symptoms inventory (NPSI) the day before the intervention for all patients**. The NPSI accounts for five different pain subscales: (1) burning (superficial) spontaneous pain *(*$Q1)$*;* (2) pressing (deep) spontaneous pain *(*$\frac{Q2+Q3}{2})$*;* (3) paroxysmal pain *(*$\frac{Q5+Q6}{2})$*;* (4) evoked pain *(*$\frac{Q8+Q9+Q10}{3})$*;* (5) paresthesia/dysesthesia $\left( \frac{Q11+Q12}{2} \right)$*.* Taken from [1].

[1] D. Bouhassira *et al.*, ‘Development and validation of the Neuropathic Pain Symptom Inventory’, *Pain*, vol. 108, no. 3, 2004, doi: 10.1016/j.pain.2003.12.024.
